# SyStemCell: A Database Populated with Multiple Levels of Experimental Data from Stem Cell Differentiation Research

**DOI:** 10.1371/journal.pone.0035230

**Published:** 2012-07-13

**Authors:** Jian Yu, Xiaobin Xing, Lingyao Zeng, Jiehuan Sun, Wei Li, Han Sun, Ying He, Jing Li, Guoqing Zhang, Chuan Wang, Yixue Li, Lu Xie

**Affiliations:** 1 Shanghai Center for Bioinformation Technology, Shanghai, China; 2 Key Lab of Systems Biology, Shanghai Institutes for Biological Sciences, Chinese Academy of Sciences, Shanghai, China; 3 Huazhong Science and Technology University, Wuhan, Hubei, China; 4 Tongji University, Shanghai, China; University of California Riverside, United States of America

## Abstract

Elucidation of the mechanisms of stem cell differentiation is of great scientific interest. Increasing evidence suggests that stem cell differentiation involves changes at multiple levels of biological regulation, which together orchestrate the complex differentiation process; many related studies have been performed to investigate the various levels of regulation. The resulting valuable data, however, remain scattered. Most of the current stem cell-relevant databases focus on a single level of regulation (mRNA expression) from limited stem cell types; thus, a unifying resource would be of great value to compile the multiple levels of research data available. Here we present a database for this purpose, SyStemCell, deposited with multi-level experimental data from stem cell research. The database currently covers seven levels of stem cell differentiation-associated regulatory mechanisms, including DNA CpG 5-hydroxymethylcytosine/methylation, histone modification, transcript products, microRNA-based regulation, protein products, phosphorylation proteins and transcription factor regulation, all of which have been curated from 285 peer-reviewed publications selected from PubMed. The database contains 43,434 genes, recorded as 942,221 gene entries, for four organisms (Homo sapiens, Mus musculus, Rattus norvegicus, and Macaca mulatta) and various stem cell sources (e.g., embryonic stem cells, neural stem cells and induced pluripotent stem cells). Data in SyStemCell can be queried by Entrez gene ID, symbol, alias, or browsed by specific stem cell type at each level of genetic regulation. An online analysis tool is integrated to assist researchers to mine potential relationships among different regulations, and the potential usage of the database is demonstrated by three case studies. SyStemCell is the first database to bridge multi-level experimental information of stem cell studies, which can become an important reference resource for stem cell researchers. The database is available at http://lifecenter.sgst.cn/SyStemCell/.

## Introduction

Stem cells are of great interest to the biomedical research community due to their differentiation pluripotency and capability of unlimited self-renewal. Elucidation of the underlying molecular mechanisms of stem cell differentiation could contribute to the advancement of cell-based regenerative medicine [1]. In the last decade, many large-scale experiments have been performed to investigate the process of stem cell differentiation from different perspectives, and abundant data have been generated. DNA CpG 5-hydroxymethylcytosine/methylation (5 hmC/5 mC) and histone modification have been proved to play crucial roles in regulating stem cells during differentiation [2], [3], [4]. Transcriptome profilings and mass spectrometry analyses have revealed characteristic gene/miRNA expression patterns and protein abundance/kinase-substrate dynamics which are specific to some stem cell types and their differentiated counterparts [5], [6], [7], [8]. Transcription factors (TF) such as Pou5f1 (Oct4), Sox2 and Nanog have always been considered essential for establishing the regulatory networks that define and maintain the undifferentiated state of stem cells [9], [10].

**Figure 1:**
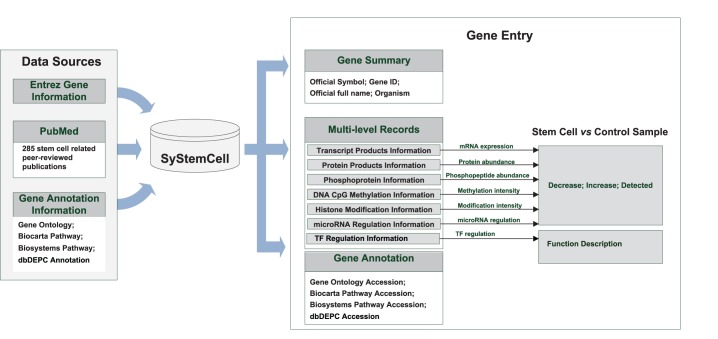
Pipeline of data collection, curation and recording in SyStemCell.

However, most experimental data generated by recent modern technology for different levels of regulation and different stem cell types are still scattered in individual published papers, as included results or even as supplementary materials. Given that recent evidence indicates that different levels of regulatory mechanisms could interact to orchestrate the complex differentiation process [11], [12], [13], a unifying resource with a comprehensive collection of currently available multi-level, multi-organism stem cell data could be of great value to allow for cross-referencing of such orchestration, and thus promoting stem cell related research.

Several pioneer databases have been developed to collect stem cell-related information; many of them focus on single-level experimental data from limited studies. BloodExpress (http://hscl.cimr.cam.ac.uk/bloodexpress/index.html) stores 271 gene expression profiles derived from 15 distinct studies on mouse immature stem cells, intermediate multipotent progenitors and mature blood cells [14]. FunGenES (http://biit.cs.ut.ee/fungenes/) covers eleven datasets of mRNA expression profiles focusing on mouse ES cells [15]. Besides the most widely studied expression profiles, some databases provide other kinds of information. CELLPEDIA (http://cellpedia.cbrc.jp/), a repository for human cell studies and differentiation analyses, provide cell location and taxonomy information other than compiling gene expression data derived from journal papers [16]. StemDB (http://www.stemdb.org/stemdb/) which was mainly designed for stem cell project management, contains stem cell-relevant information on antibodies, markers, primers other than large-scale mRNA expression data. Recently databases curating data from more than one regulatory level start to emerge, but only with limited stem cell types. For instance, UESC is a database for urologic epithelial stem cells with gene expression and immunohistochemistry images [17] (http://scgap.systemsbiology.net/). The last on the list is ESCDb (http://biit.cs.ut.ee/escd/help.html), which gathers ChIP and microarray experiments with a focus on pluripotency associated TFs involved in human and mouse ES and carcinoma cells [18]. Compared to UESC, ESCDb offers a summarized view of its multiple-level data collection, but the web page does not support data browsing and its latest datasets are now out of date (lastly updated two years ago).

Therefore, we have developed SyStemCell, a database populated with seven levels of experimental data manually curated from 285 carefully selected publications from PubMed. Its data collection ranges from DNA CpG 5-hydroxymethylcytosine/methylation (5 hmC/5 mC), histone modification, transcript products, microRNA-based regulation, protein products, phosphorylation proteins and TF regulation, covering diverse stem cell types from four organisms (*Homo sapiens*, *Mus musculus*, *Rattus norvegicus*, and *Macaca mulatta*). An online analysis tool is also integrated to mine potential relationships among different regulation levels and possibly formulate new hypothesis. Besides, by comparing data of human and mouse available in the download section, a co-regulatory network is investigated which is conserved in these two species. All these characteristics render SyStemCell a most comprehensive and up-to-date resource for stem cell research currently. It would provide a basic platform for users to extract relationships suggested by the multi-source data and should contribute to more in-depth understanding of stem cell biology.

**Figure 2:**
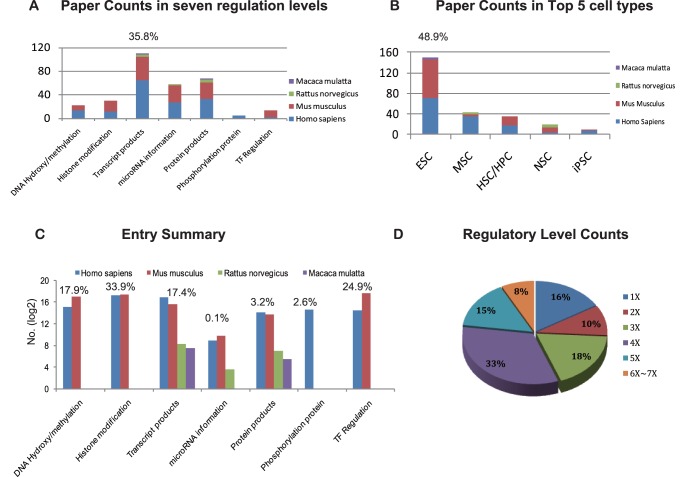
Database content of SyStemCell. (A) Summary of original papers on seven levels of regulation, where transcription products possess the largest proportion of all recorded papers in SyStemCell. (B) Summary of Top 5 stem cell types from original papers, where the proportion of ESC (Embryonic Stem Cells) ranks the first. MSC, Mesenchymal Stem Cells; HSC/HPC, Hematopoietic Stem/Progenitor Cells; NSC, Neural Stem Cells and iPSC, induced Pluripotent Stem Cells. (C) Summary of entry across seven regulatory levels. The entry counts are log2 transformed for each level. (D) Pie plot of regulatory levels occupied by all 43,434 genes in SyStemCell.

## Methods

### Data Collection and Curation

A semi-automatic method was employed to collect and curate multiple levels of original qualitative and quantitative stem cell experimental data from peer-reviewed publications in PubMed (Figure 1), as follows:

- PubMed was automatically surveyed for large-scale experiments using the keyword “stem cell” along with level-specific keywords for the time period June 2000 to June 2011. The level-specific keywords included “DNA methylation”, “DNA 5-hydroxymethylcytosine”, “histone modification” and “ChIP-Seq” for epigenetic modification; “transcription profile”, “expression profile”, “transcriptome”, “transcriptomics”, “RNA-Seq” and “microarray” for mRNA expression; “microRNA” for microRNA regulation; “proteome”, “proteomics”, and “mass spectrometry” for protein abundance; “phosphorylation” and “phosphoproteome” for protein phosphorylation information; “ChIP-Chip”, “ChIP-Seq” and “transcription factor” for transcriptional regulation. In addition, PubMed was searched for specific studies on stem-cell master genes (e.g., Pou5f1) with low-throughput experimental results (e.g., Western blot, real-time PCR, bisulfite sequencing).
- To ensure data availability and quality, the original data in retrieved papers were manually checked, for the following points of concern: (1) whether the experimental cell type was defined as stem cell (e.g., excluding precursors); (2) whether the experimental data was included in original paper of available in supplementary information; (3) whether experimental design relevant to the data generation was provided. Based on these criteria, 285 publications were selected, of which 22 papers were related to DNA CpG 5 hmC/5 mC, 30 to histone modification, 109 to mRNA expression, 58 to microRNA regulation, 68 to protein abundance, 5 to protein phosphorylation and 14 to TF regulation (Table S1, one paper may cover two or more regulatory levels). The data for both large-scale and low-throughput experiments were strictly curated as raw gene entries before being deposited into SyStemCell. The items recorded for each raw gene entry at each regulatory level include: original gene/protein accession number, stem cell type, control sample type, treatment used to induce stem cell differentiation (if data available), regulatory state in stem cell sample compared to control sample, and PubMed accession number. Statistical cutoffs for mRNA/miRNA/protein detected and/or differentially expressed, specific experimental operation platforms, and other related original information in each publication were also extracted and recorded along with gene entries (Table S2).
- The original gene/protein accession numbers in raw gene entries were derived from various data sources, including Entrez Gene [19], UniGene (http://www.ncbi.nlm.nih.gov/unigene), GeneBank [20], NCBI Refseq [21], UniProt [22], and Ensembl [23]. To cross-link the multi-level data in SyStemCell, all original accession numbers are referenced to Entrez Gene.
- Gene annotation information was extracted from the Gene Ontology database [24], Biocarta Pathway (http://www.biocarta.com/), Biosystems Pathway [25] and dbDEPC [26]. Biocarta Pathway contains signaling pathway information in human and mouse while Biosystems Pathway defines biosystems consisting of interacting genes, proteins, and small molecules (http://www.ncbi.nlm.nih.gov/biosystems). dbDEPC is an in-house database of differentially expressed proteins in human cancers, which might allow a quick check of tumor relevance for genes identified in stem cell research.

**Figure 3:**
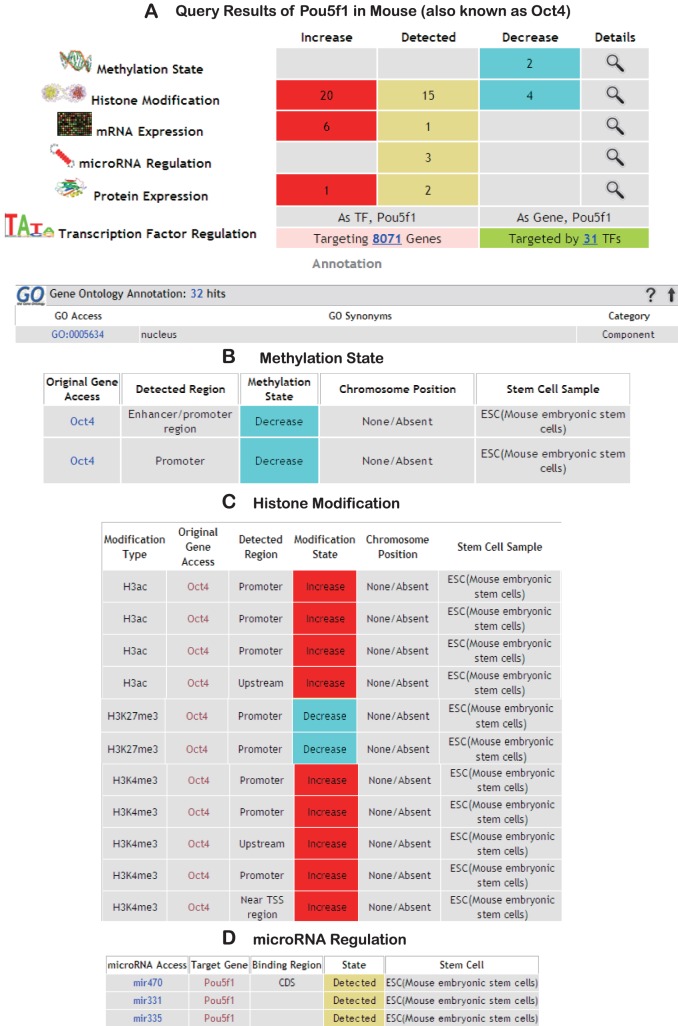
Queries retrieved from SyStemCell, using mouse gene “Pou5f1” (Oct4) as an example. (A) Multi-level summary page and external annotation (only partial displayed). (B) DNA CpG Methylation information. (C) Histone modification information (only partial displayed) and (D) microRNA regulation information.

### Database Construction

SyStemCell consists of a relational database and a dynamic web interface, implemented using Mysql Server Edition 5.0 and configured on a running RedHat Linux Server. The web interface is implemented with JSP technology with AJAX using an Apache Tomcat 6.0 Server. The online analysis tools, including co-localization analysis and venn-diagram plotting, are developed with R (http://www.r-project.org/).

#### Database availability

SyStemCell can be accessed via http://lifecenter.sgst.cn/SyStemCell/. All data in SyStemCell are freely available through the download page http://lifecenter.sgst.cn/SyStemCell/Download.jsp.

**Figure 4:**
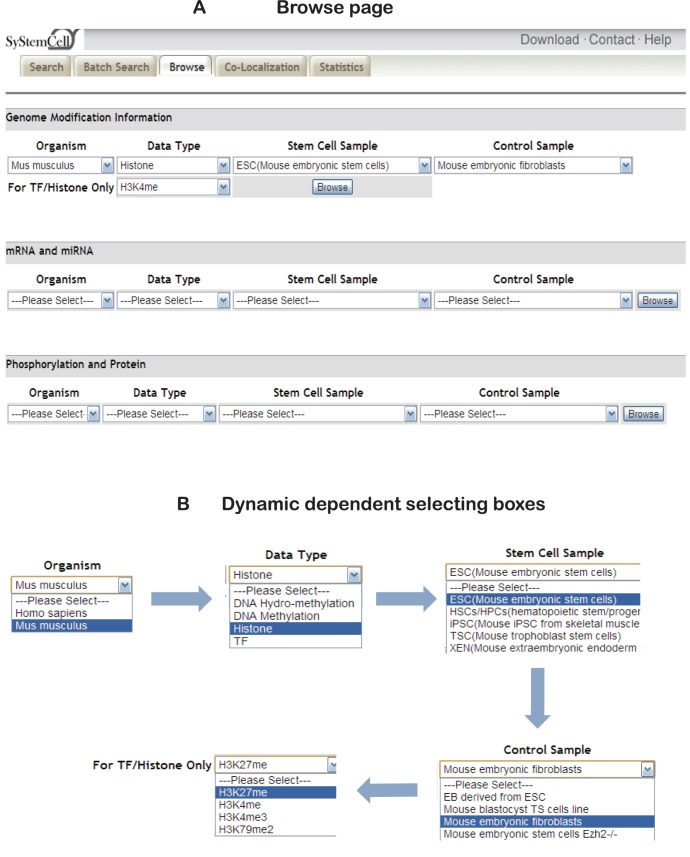
Browse page and dynamic selecting box. (A) Browse page for seven levels of regulatory information in SyStemCell. (B) Dynamic selecting box (using histone modification H3K27me3 in mouse ES and fibroblasts cells as an example). “Child” boxes are only displayed when their “Parent” boxes are selected.

**Figure 5:**
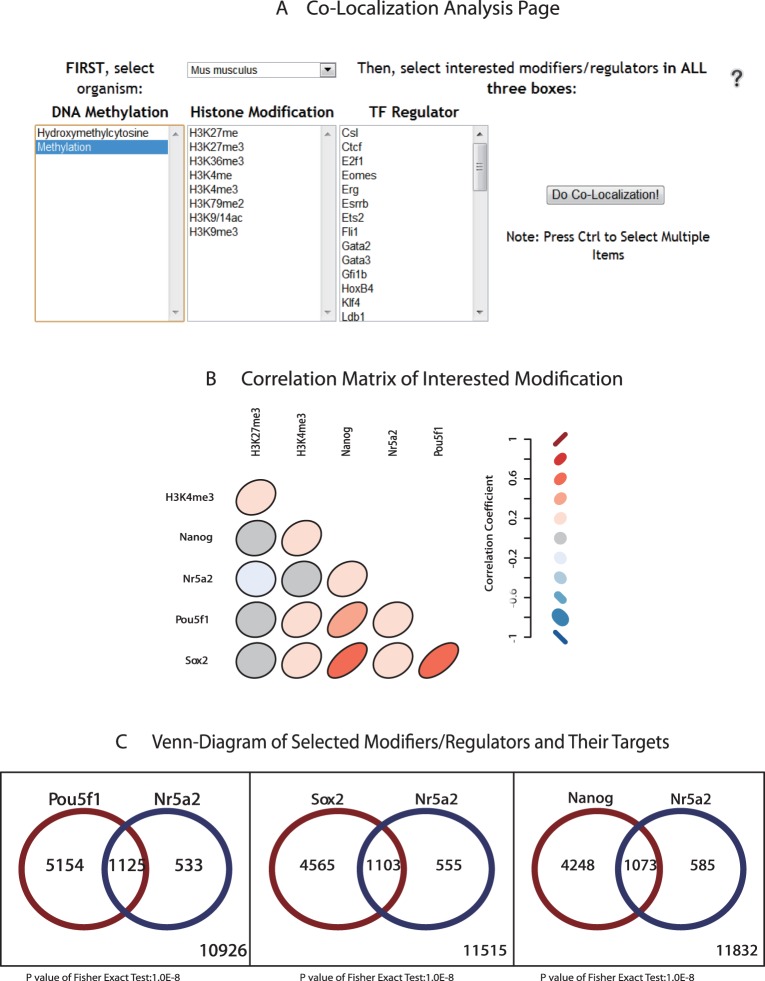
Co-Localization analysis page and example. (A) Analysis can be carried in two organisms (human and mouse) and three regulation levels (CpG hydroxy/methylation, histone modification and transcription factor binding) (B) Correlation matrix created by selecting interested modifiers/regulators (Pou5f1, Nr5a2, Sox2, Nanog, H3K4me3 and H3K27me3) in mouse. The color of red and shape close to slash indicate more positive correlation, while the color of blue and shape close to backslash indicate negative correlation, and the color of grey and shape like circle indicate no correlation. (C) Venn-diagram of Pou5f1 targeted genes and Nr5a2 targeted genes. Gene list in each part of the plot can be downloaded separately to run enrichment analysis in DAVID.

## Results

### Database Content

Currently, SyStemCell covers four organisms (*Homo sapiens*, *Mus musculus*, *Rattus norvegicus*, and *Macaca mulatta*) and diverse stem cell types, including ES cells, hematopoietic stem/progenitor cells (HSC/HPC), mesenchymal stem cells (MSC), induced pluripotent stem cells (iPSC), neural stem cells (NSC), cancer stem cells, and others. Regarding cell type and data type in publications, ES cell related studies (48.9%) and transcript-level data (35.8%) constitute the most abundant knowledge in stem cell research (Figure 2A–B). However, as for entry count, DNA 5 hmC/5 mC, histone modification and TF regulation now form the predominant proportion of SyStemCell (76.7%), due to the explosion of ChIP-Seq technology.

The database now contains information covering seven levels of stem cell gene regulation, including DNA CpG 5 hmC/5 mC (168,291 entries, 27,645 for 5 hmC and 140,646 for 5 mC), histone modification (319,496 entries), mRNA expression (164,089 entries), microRNA-based regulation (1,412 entries), protein abundance (30,299 entries), protein phosphorylation (24,360 entries) and TF regulation (234,274 entries) (Figure 2C). In total, 43,434 Entrez genes are recorded in SyStemCell; of these, 36,385 genes (84%) show more than one level of regulation, and 24,196 genes (56%) demonstrate four to seven levels of regulation (Figure 2D). Please note that regulatory state is denoted as “increase” (hypermethylation/histone modification/phosphorylation/and up-regulated in transcript products, miRNA expression and protein abundance), “decrease” (hypomethylation/without histone modification/without phosphorylation/and down-regulated in transcript products, miRNA expression and protein abundance), when comparing stem cells with control. If the state is recorded as “detected”, it means either there were no control cells in experimental design or no statistic test (such as p-value and false discovery rate) was conducted in the original paper (Figure S1: A–D). The only exception which cannot be denoted as “increase”, “decrease” or “detected” is transcription factor regulation, in which genes are only categorized into two statuses: transcription factor (TF) and TF targets (Figure S1: E).

**Figure 6:**
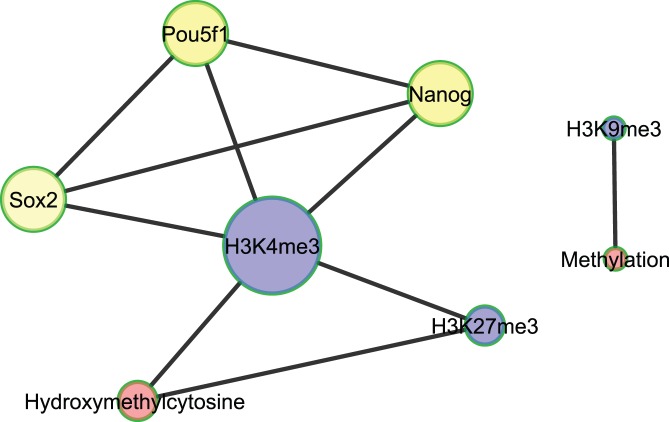
Conserved co-regulatory network in both Homo sapiens and Mus musculus species. Each interconnected edge (representing a pair of modifier/regulator) must satisfy three criteria, i.e., existed in both human and mouse, the Bonforroni adjusted p<0.001 and the intersection genes of the pair was enriched at least 2-fold. The gene symbols are shown as in Mus musculus species. The node size is in proportion to its degree and color represents different types of modifier/regulator, red, DNA hydroxy/methylation; blue, hisotone modification and yellow, transcription factor.

### Database Utility

SyStemCell provides two data-retrieving methods on its homepage. One is gene-based query, supporting Entrez gene ID, symbol, or alias. The retrieved page includes information in three sections: Gene Description, Multi-level Data visualization, and Gene Annotation. If any information about the query gene is present in the database, SyStemCell will first come up with a gene summary section, including the official gene symbol, gene ID, official full name, and organism. Next, in the multi-level visualization section, its related entries are summarized as a heatmap-like table, where the red indicates “up-regulated”, the grey “detected only” and the blue “down-regulated” (Figure 3A, with the mouse stem cell master gene “Pou5f1” as a query gene). Numbers in the table indicate the entry count for each regulation level in each state. More detailed information about each regulatory level can be viewed and downloaded in another page for further investigation through a “magnifier” bottom (Figure 3B–D). Below this part is the gene annotation section, providing annotation information from Gene Ontology, Biocarta Pathway, Biosystems Pathway and dbDEPC. Additionally, in the page of mRNA expression and protein abundance, a brief summary of experimental record information is supplied, covering related platform, preprocessing method and filtering condition (Figure S1F). All the available annotations are hyperlinked to the original page in their corresponding databases (GO, dbDEPC, NCBI and Biocarta).

SyStemCell also allows for stem cell-specific data browsing via the ‘browse’ page (Figure 4A). Users can browse by organism, level of regulation, stem cell type, and/or control sample. Powered by Ajax technology, dynamic dependent box is implemented in this page to avoid null hits during browsing. When a selection is made in a “Parent” box (e.g., mouse ES cells as “Stem Cell Sample”), it allows a “Child” list box to return matched information (e.g., embryonic fibroblasts as “Control sample” of ES cells) available in the database (Figure 4B). After all boxes are selected, the retrieved page will display related information and provide another standalone page similar to Figure 3B–D for users to download these results.

**Table 1:** Nodes with high coreness in combinatorial TF-miRNA network of mouse ESC.

Name	Coreness	core TFs in ESC*
Klf4	16**	YES
Tcfcp2l1	16	YES
Sall4	16	YES
Pou5f1	16	YES
Nipbl	16	NO
Nanog	16	YES
Mycn	16	YES
Sox2	16	YES
E2f1	16	YES
Tbp	16	NO
Smc1a	16	NO
Med12	16	NO
Med1	16	NO
Esrrb	16	YES
Ctcf	16	YES
Smc3	16	NO
Mycn	16	YES
Stat3	16	YES
Zfx	15	YES
Zic3	14	NO
Tcfap2c	14	NO
Smad1	14	YES
Ldb1	14	NO
Smarca4	13	NO
Sall4b	10	NO
Meis1	10	NO
mmu-miR-762	10	–
mmu-miR-705	10	–
mmu-miR-455-5p	10	–
mmu-miR-34a-5p	10	–
mmu-miR-1958	10	–
mmu-miR-190-5p	10	–

* ESC, Embryonic Stem Cells.

** Only the nodes with coreness larger than 10 are displayed in the table.

**Figure 7:**
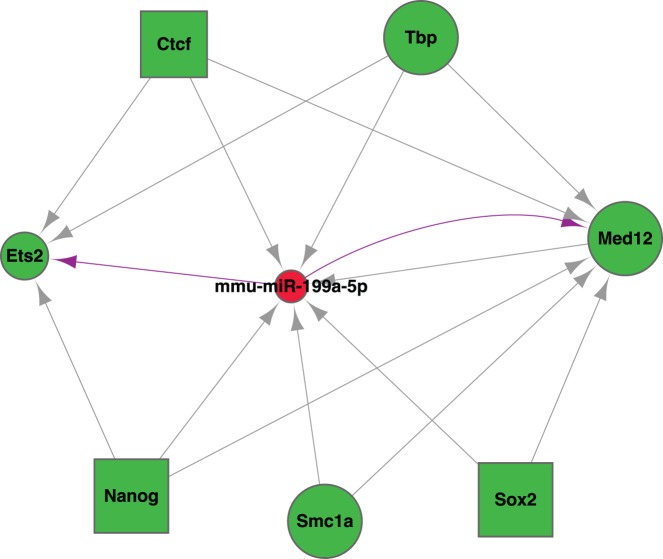
Motif patterns in the mouse ESC combinatorial network. Green nodes represent TFs, and red nodes represent miRNAs. Nodes in rectangle shape are ESC core TFs according to literatures. All the edges are retrieved from SystemCell except those in purple, which are supplemented by predicted miRNA-target relationships.

### Co-Localization Analysis Tool

It is now believed that the ‘stemness’ state of stem cell is regulated by the orchestration of transcription regulation network as well as a set of ‘chromatin signatures’ that support an environment maintaining self-renewal and that are permissive for differentiation [27]. SyStemCell therefore implements an online analysis tool to help researchers investigate the correlation among three important regulation levels: DNA 5 hmC/5 mC, histone modification and transcription factor regulation (Figure 5A). A lower triangular matrix consisted of ellipses with different colors can be plotted in the Co-localization Analysis page, after selecting interested epigenetic modifications such as H3K4me3, H3K27me3 (histone modifications), and Nr5a2, Pou5f1 (also known as Oct3/4), Sox2 and Nanog (transcription factors) in mouse genome (Figure 5B). Each ellipse represented a spearman correlation coefficient between two modifiers/regulators, which was conducted by following steps: First, the presence of each modifier/regulator in mouse/human genome was summarized, where 1 represents detected and 0 represents none. Next the “0” or “1” was composed into a vector in the order of gene names and spearman correlation coefficients were calculated between each modification pair. Finally a graphical display of correlation matrix was plotted, where color of red and ellipse shaping close to slash indicate more positive correlation, color of blue and ellipse shaping close to backslash indicate negative correlation, and color of grey and shaping circle indicate no correlation. To further demonstrate the intersection of regulated genes by interested co-localized pairs, and to test whether the intersection is random, SyStemCell also provides an online Venn-Diagram plotting tool (Figure 5C) that can be followed by enrichment analysis via DAVID [28].

### Case Studies of Utilizing the Database and the Co-localization Tool

To illustrate applications of SyStemCell, here we propose three examples in three levels: single-gene search and result display, co-localization of selected group of modifications and TFs, co-regulatory network that conserves across species by comparing whole datasets from different species.

A prominent mouse stem cell master gene, Pou5f1, critical for early embryogenesis and for ES cell pluripotency [29], [30], is recorded with six levels of regulation in SyStemCell (Figure 3A). The gene query results show that mRNA expression and protein abundance are significantly increased in stem cells than their differentiated counterparts, which can be confirmed in many related experiments across different regulation levels. The increase could be associated with the following changes, detailed in Figure 3B–D: 1) decrease in DNA CpG methylation intensity in the promoter region, which could facilitate gene expression [31], [32], 2) increase in the histones H3ac, H3K4me3, and decrease in H3K27me in the upstream/promoter region, which also could influence mRNA expression level [33], [34], [35], and/or 3) microRNA-induced degradation of Pou5f1, as suggested by several experiments [36], [37].

Second, the potential usage of the co-localization analysis tool in SyStemCell is illustrated in Figure 5B, from two perspectives. Firstly, significant co-localization patterns among Oct4 (Pou5f1), Sox2 and Nanog (OSN) are observed, in good agreement with the findings that these three factors form the core of a transcription factor network that act synergistically for ES cell pluripotency and self-renewal both in human and mouse [38], [39], [40]. Secondly, the co-localization pair of H3K4me3 and H3K27me3 (Figure 5B) supports previous discoveries that they are the most studied bivalent modification contributing to development control of ES cells [4], [41]. Besides conforming to existed knowledge, this analysis tool may also provide new insights to formulate hypotheses. For example, Figure 5B shows a correlation between different regulation levels: H3K4me3 and OSN genes. Their interconnectivity remained unclear until very recently when H3K4me3 was found to interact with core transcriptional network to maintain ES cell self-renewal [42]. Another example, all OSN genes share a proportion of target genes with Nr5a2 (Figure 5B–C), suggesting Nr5a2 may bypass the need of OSN genes in iPSC derivation from somatic cell reprogramming, and this was realized experimentally by Heng et al [43] in 2010.

Finally, integrating data across different species to reveal evolutionarily conserved regulatory patterns in stem cells is always of great interest. Here, by combining epigenetic modification (including transcription regulation) data in both Mus Musculus and Homo Sapiens, a co-regulatory network was extracted to represent a brief overview of transcription regulation and epigenetic modification that existed or ‘conserved’ in both species (Figure 6). The co-regulatory network was plotted by selecting candidate pairs satisfying the following three criteria in co-localization analysis: i) the candidate pair existed in both human and mouse; ii) the Bonforroni adjusted p value of spearman correlation was below 0.001 and iii) the intersection genes of the pair was enriched 2-fold than random expectation. In this co-regulatory network, notably H3K4me3 is the hub with the largest degree, showing its multi-faceted roles in mediating DNA 5 hmC (Hydroxymethylcytosine) [44], histone modification (H3K27me3) [45] and TF targeting (OSN: Sox2, Pou5f1 and Nanog) [46] in a conserved approach in both Homo sapiens and Mus musculus species. The bivalent modification between H3K4me3 and H3K27me3 and the interaction of H3K4me3 with OSN were also identified in the second case-study(the above paragraph).

Another intriguing finding shown in the co-regulatory network is that 5 hmC, a previously unappreciated modification of DNA but now considered the sixth base of genome [47], connected to both transcription-active modification marker H3K4me3 and repressive marker H3K27me3. Although the detailed mechanisms and function of 5 hmC remain enigmatic, it has been implicated that 5 hmC plays a dual role in transcription regulation [48]. When modified by H3K4me3, it may contribute to maintaining a more accessible chromatin structure to facilitate TF binding; on the other hand, when connected to the trimethylation of H3K27(H3K27me3) it may help the generation of heterochromatin, thus preventing TF binding [49]. Together, the conserved relations of 5 hmC with H3K4me3 and H3K27me3 suggest that 5 hmC may be essential in stem cell transcription regulation, by associating with a ‘poised’ chromatin configuration. Lastly the co-localized pair of H3K9me3 and methylation is also conserved in both Homo sapiens and Mus musculus species, which has been indicated as an ES-specific silencing mechanism to protect the stability of genome from the threat of endogenous retroviruses and retrovirus-like elements [50].

### Study of Combinatorial Network Including TFs and miRNAs in ESC

The roles of miRNAs are emerging in the establishment and maintenance of ESC identity [51]. Investigation into the topology and properties of the combinatorial network including TFs and miRNAs is helpful for us to understand the interplay between these two types of transcriptional regulators [52]. Here we propose a simple combinatorial network analysis in the context of mouse embryonic stem cells (ESC) in order to show the rationale and usefulness of our database in a specific topic research related to ESC.

Construction and validation of the mouse ESC network: Our database included TF-TF and TF-miRNA regulatory relationships in mouse embryonic stem cells, while miRNA-TF relationships were not included. In order to supplement the miRNA-TF relationships, we resorted to miRNA target prediction algorithms, miRanda [53] and TargetScan [54]. Then a combinatorial regulatory network in mouse embryonic stem cells was constructed and validated by the classic transcriptional regulators in ESC (Figure S2). Based on published studies [10],[55], a list of 21 transcriptional regulators implicated in the ES cells were collected. Of the 21 core regulators in ESC, 14 could be mapped to the regulatory relationships in our database (3-rd column in Table 1).

Identification of mouse ESC-related miRNAs through network analysis: Coreness of nodes was calculated as a description of clustering structure of a network graph [56]. It turned out that most nodes with high coreness (clustering together with high degrees) were the ESC core TFs, and 6 miRNAs ranked as high-coreness nodes as well (Table 1). Motif patterns such as feed-forward loop and feed-back loop [52], [57] were also investigated (Figure 7). Among the one feed-back loop and 8 feed-forward loops, mmu-miR-199a-5p played as an important miRNA regulator in concert with TFs in mouse ESC.

## Discussion

Until now, a large proportion of gene information across diverse regulatory levels and species are still scattered among literatures in the field of stem cell research, and a database collecting and integrating such information is in great need. To address this issue, SyStemCell, a database populated with multiple levels of experimental data from stem cell differentiation research, was established and now available for data query, browse, analysis and accession to other related resources. In the section of case study, the first example (shown by Pou5f1) illustrated how SyStemCell can provide a comprehensive picture in diverse regulatory levels of any stem cell related gene. In total, 36,385 genes (84%) can be found with more than one level of regulation information recorded in SyStemCell; these records could be cross-referenced to help promote understanding of gene regulation mechanisms in stem cell.

With the explosion of ChIP-Sequencing technology, the entry counts of epigenetic modification and TF regulation go far beyond those in transcripts and protein products, forming the predominant proportion of SyStemCell. Therefore, a unique co-localization analysis tool aimed to investigate potential relationship among DNA CpG 5 hmC/5 mC, histone modification and TF regulation has been developed and deployed in SyStemCell, which may help mark out substantial biological effectors and suggest underlying molecular circuit in the complex progress of stem cell self-renewal and differentiation [58], [59], [60]. Such examples include the prevalent bivalent modification of H3K4me3/H3K27me3 and the core OSN transcription network in stem cell, as well as the potential effect of Nr5a2 in cell reprogramming. Furthermore, after combining data from Homo sapiens and Mus musculus, the pivotal role of H3K4me3 and dual function of 5 hmC were emphasized from an evolutionarily conserved viewpoint, highlighting the potential value of further stem cell research with the aid of data integration available in SyStemCell.

Mouse embryonic stem cells (ESC) are populated with the most information at transcription expression levels: mRNA and miRNA, and TF-TF and TF-miRNA regulatory relationships were also annotated in the database. Incorporating such abundant information, and making use of other bioinformatics strategies such as miRNA targets prediction, network topology analysis, we were able to show even more complicated research study based on SyStemCell, that is the constructing of a combinatorial network including TFs and miRNAs as regulators. Of the 21 core regulators in mouse ESC, 14 could be mapped to the regulatory relationships in our database. Motif patterns such as feed-forward loop and feed-back loop were also investigated, and mmu-miR-199a-5p was found to act as an important miRNA regulator in concert with TFs in mouse ESC.

Overall, SyStemCell has been constructed in the hope of providing a comprehensive stem cell library with more information of diverse regulatory levels and species than existed databases before. Other than using SyStemCell as a data-depositing library only, through cross-referencing and elaborating Co-localization Analysis Tool provided in the webpage, or through integrating large datasets in specific stem cell types, which were all examplified in this paper, users may very well likely to be able to research on certain interested topics in stem cell biology field with the help of SyStemCell.

### Supplementary Data

Supplementary data are available Online.

## Supporting Information

Figure S1 **Summary of entry state according to regulatory state across six levels in four organisms.** (A) Homo sapiens, (B) Mus musculus, (C) Rattus norvegicus and (D) Macaca mulatta. The only exception is transcription factor (TF), where gene is categorized into two states, TF and TF target (E). (F) Experimental information related to mRNA expression and protein abundance was embedded in supplied in a standalone web page.
(EPS)

Figure S2 **Overview of the mouse ESC combinatorial network.** Size of each node is in proportion to its coreness. Green nodes represent TFs, and red nodes represent miRNAs. Nodes in rectangle shape are ESC core TFs according to literatures. All the edges are retrieved from SystemCell except those in purple, which are supplemented by predicted miRNA-target relationships.
(EPS)

Table S1 **List of 285 peer-reviewed publications in PubMed, from which the data in SyStemCell were curated.**
(XLS)

Table S2 **List of experimental information extracted from 285 peer-reviewed publications according to seven levels of regulation.** It is organized in six sheets (“protein product” and “phosphoprotein” were combined together in one sheet).
(XLS)
